# Characteristics of lipid profiles and lipid control in patients with diabetes in a tertiary hospital in Southwest China: an observational study based on electronic medical records

**DOI:** 10.1186/s12944-018-0945-8

**Published:** 2019-01-12

**Authors:** Qingtao Hou, Chuan Yu, Sheyu Li, Yun Li, Rui Zhang, Tao Zheng, Yi Ma, Miye Wang, Na Su, Ting Wu, Zhiwen Liu, Xia Sheng, Nan Li, Guanjian Liu, Yong Huang, Ting Xu, Xin Sun, Haoming Tian

**Affiliations:** 1 West China School of Medicine, Sichuan University, Chengdu, 610041 China; 2 Department of Geriatrics, The First Affiliated Hospital of Chongqing Medical University, Chongqing, 400016 China; 3 Department of Health-Related Social and Behavioral Science, West China School of Public Health, Sichuan University, Chengdu, 610041 China; 4 CREAT Group, Chinese Evidence-Based Medicine Center, West China Hospital, Sichuan University, Chengdu, 610041 China; 5 Department of Endocrinology and Metabolism, West China Hospital, Sichuan University, Chengdu, 610041 China; 6 Department of Endocrinology and Metabolism, Deyang People’s Hospital, Deyang, 618000 China; 7 Health Informatics Center, West China Hospital, Sichuan University, Chengdu, 610041 China; 8 Department of Pharmacy, West China Hospital, Sichuan University, Chengdu, 610041 China; 9 Epidemiology Asia Pacific Unit, Merck Research Laboratories, Merck Sharp and Dohme Corp, Chengdu, 610041 China; 10 Department of Pharmacoepidemiology, Merck Research Laboratories, Merck Sharp and Dohme Corp, Chengdu, 610041 China; 11 Informatics IT Asia Pacific Unit, Merck Research Laboratories, Merck Sharp and Dohme Corp, Beijing, 100000 China

**Keywords:** Diabetes, Electronic medical record, Inpatient, Lipids

## Abstract

**Background:**

Diabetes is often accompanied by dyslipidemia. Lipid control is very important in the management of diabetes. There are limited real world data on the lipid control in diabetic inpatients in southwest China.

**Methods:**

An observational study was conducted to assess the characteristics of lipid profiles and lipid control. Diabetic patients from February 2009 to December 2013 at West China Hospital of Sichuan University were identified.

**Results:**

A total of 56,784 inpatients were included and 85.9% of them had at least one lipid panel. The proportions of inpatients with optimal low-density lipoprotein cholesterol (LDL-C) level (< 2.59 mmol/L), optimal triglyceride (TG) level (< 1.70 mmol/L), optimal high-density lipoprotein cholesterol (HDL-C) level (men ≥1.04 mmol/L; women ≥1.30 mmol/L) and optimal non-high-density lipoprotein cholesterol (non-HDL-C) level (< 3.37 mmol/L) were 61.1, 64.6, 49.9 and 64.5%, respectively. Only 23.1% of inpatients obtained optimal levels for all the above four lipid parameters. Of diabetic inpatients with ischemic heart disease, the proportions of inpatients with optimal LDL-C level (< 1.81 mmol/L), optimal TG level (< 1.70 mmol/L), optimal HDL-C level (men ≥1.04 mmol/L; women ≥1.30 mmol/L) and optimal non-HDL-C level (< 2.59 mmol/L) were 38.0, 66.3, 48.1 and 48.7%, respectively. Of diabetic inpatients with cerebrovascular disease, the proportions were 28.3, 64.8, 49.9 and 38.1%, respectively. Older people and men were more likely to obtain optimal lipid levels. However, inpatients between 46 and 64 years were least likely to obtain optimal LDL-C levels.

**Conclusions:**

The lipid control of diabetic inpatients in southwest China is worrisome. Individualized strategies of lipid management should be taken to bridge the gap between the recommendations of clinical guidelines and the real situation of clinical practice.

**Electronic supplementary material:**

The online version of this article (10.1186/s12944-018-0945-8) contains supplementary material, which is available to authorized users.

## Background

Diabetes, with an increasing prevalence, has become one of the most important chronic diseases. The International Diabetes Federation (IDF) data shows that the prevalence of diabetes in 2013 is 8.3% globally, affecting 382 million adults, and this number is expected to climb to 10.1%, affecting 592 million adults by 2035. Diabetes has become an epidemic in China. The total number of adults between 20 to 79 years old with diabetes in China in 2013 is 98.4 million and in 2035 this number is expected to rise to 142.7 million [1, 2]. Diabetes has resulted in a huge economic burden for the whole society [3, 4].

Diabetes is often accompanied by dyslipidemia. The diagnostic criteria and treatment targets of dyslipidemia in diabetic patients are different from those in the general population [5]. The National Cholesterol Education Program-Adult Treatment Panel III (NCEP-ATP III) guideline recommends that low-density lipoprotein cholesterol (LDL-C) is the primary goal in the management of dyslipidemia and coronary heart disease (CHD). And LDL-C should be controlled below the level of 2.59 mmol/L (100 mg/dL) [6]. The American Diabetes Association (ADA) guideline recommends that the levels of LDL-C, triglyceride (TG) and high-density lipoprotein cholesterol (HDL-C) should be controlled below 2.59 mmol/L, 1.70 mmol/L and 1.04 mmol/L (1.30 mmol/L for women) for diabetic patients, respectively. Non-high-density lipoprotein cholesterol (non-HDL-C) is suggested as the secondary goal for the treatment of CHD and should be controlled below the level of 3.37 mmol/L. Among diabetic patients with CHD, the LDL-C and non-HDL-C levels should be controlled below 1.81 mmol/L and 2.59 mmol/L, respectively [7, 8]. Diabetic dyslipidemia increases the risk of cardiocerebrovascular diseases. Lipid control is very neccessary during the whole process of diabetes management.

At present, there is a lack of real world data on the characteristics of lipid profiles in diabetic inpatients in China. And there is limited evidence on whether dyslipidemia is adequately managed or not for patients with diabetes in the real-world practice. Thus, we conducted this real world study based on electronic medical record (EMR) system from a single tertiary hospital in southwest China to assess the characteristics of lipid profiles and the control of dyslipidemia in Chinese diabetic inpatients.

## Methods

### Study subjects and data collection

This was a single center, cross-sectional study. The study design was previously described elsewhere [9]. Diabetic patients from February 2009 to December 2013 at West China Hospital of Sichuan University were included. Diabetes was identified as International Classification of Diseases (ICD-10) codes E10-E14 and O24 at discharge. The demographic characteristics, diagnosis information at discharge, laboratory tests at admission and treatment records during the whole hospitalization were collected.

### Lipid levels categorization

Lipid levels at admission were grouped based on the NCEP-ATP III guideline. LDL-C levels were categorized into the five following groups: < 2.59 mmol/L, ≥2.59 mmol/L, ≥3.37 mmol/L, ≥4.14 mmol/L, ≥4.92 mmol/L. TG levels were grouped into four layers: < 1.70 mmol/L, ≥1.70 mmol/L, ≥2.26 mmol/L, ≥5.65 mmol/L. HDL-C levels were divided into three categories: < 1.04 mmol/L for men (< 1.30 mmol/L for women), ≥1.04 mmol/L for men (≥1.30 mmol/L for women), ≥1.55 mmol/L for men (≥1.81 mmol/L for women). Non-HDL-C levels were categorized into four groups: < 3.37 mmol/L, ≥3.37 mmol/L, ≥4.14 mmol/L, ≥4.92 mmol/L. Non-HDL-C was computed by the following formula: non-HDL-C = total cholesterol (TC) - HDL-C. LDL-C < 2.59 mmol/L, TG < 1.70 mmol/L, HDL-C ≥ 1.04 mmol/L (1.30 mmol/L for women) and non-HDL-C < 3.37 mmol/L were defined as “optimal levels”.

### Co-morbidity identification

Co-morbidities were identified according to the ICD-10 codes at discharge. Ischemic heart disease (IHD) was identified as I20-I25. Cerebrovascular disease (CVD) was identified as I60-I69 and G45. Chronic kidney disease (CKD) was identified by the following criteria: (1) the last estimated glomerular filtration rate (eGFR) < 60 mL/min/1.73 m^2^, (2) an exclusion of discharge diagnosis with acute kidney injury (ICD-10 code N17).

### Other variables

Other variables, including gender, age (≤45 years, 46–64 years, 65–79 years or ≥ 80 years) and history of smoking or alchohol were also described and analyzed in this study. History of smoking was classified into two categories: Yes (ex-smokers or current smokers), No (never smoked). History of alchohol was classified into two categories (Yes and No) similarly.

### Statistical analysis

SPSS 20.0 (IBM Co., Armonk, New York, USA) statistical software was used for the data analyses. Categorical variables were presented as frequency and percentages. Continuous variables were presented as means and standard deviations (SDs) or percentiles. Chi-square tests of proportions were used to compare the lipid category distributions in each gender and age group. Multivariate logistic regression models were used to identify the potential related factors for the lipid control.

## Results

### Baseline characteristics of inpatients with diabetes

A total of 56,784 inpatients diagnosed with diabetes from February 2009 to December 2013 were included in this study. The mean age was 63.53 ± 13.10 years. The proportion of men was 59.6%. A total of 48,700 inpatients (85.9%) had at least one lipid panel. The median levels of TC, LDL-C, TG, HDL-C and non-HDL-C for the whole population were 4.16 (3.42, 4.97) mmol/L, 2.32 (1.71, 2.97) mmol/L, 1.39 (1.00, 2.02) mmol/L, 1.13 (0.90, 1.40) mmol/L and 2.95 (2.28, 3.72) mmol/L, respectively. The proportions of patients with optimal levels of LDL-C (< 2.59 mmol/L), TG (< 1.70 mmol/L), HDL-C (men ≥1.04 mmol/L; women ≥1.30 mmol/L) and non-HDL-C (< 3.37 mmol/L) were 61.1, 64.6, 49.9 and 64.5%, respectively. Only 23.1% of patients obtained optimal levels for all the above four lipid parameters. The detailed lipid profiles and baseline characteristics are presented in Table 1.

**Table 1:** Lipid profiles and baseline characteristics of inpatients with diabetes

Variables	Mean ± SD or P50 (P25, P75) or number (percentage)
Age (years)	63.53 ± 13.10
Age group, n (%)	
< 45	5456 (9.6)
45–64	22,952 (40.4)
65–79	22,406 (39.5)
≥80	5970 (10.5)
Gender, n (%)	
Men	33,835 (59.6)
Women	22,949 (40.4)
History of alchohol, n (%)	
No	37,389 (65.8)
Yes	15,315 (27.0)
Missing	4080 (7.2)
History of smoking, n (%)	
No	33,366 (58.8)
Yes	19,576 (34.5)
Missing	3842 (6.8)
TC (mmol/L)	4.16 (3.42, 4.97)
LDL-C (mmol/L)	2.32 (1.71, 2.97)
TG (mmol/L)	1.39 (1.00, 2.02)
HDL-C (mmol/L)	1.13 (0.90, 1.40)
Non-HDL-C (mmol/L)	2.95 (2.28, 3.72)
LDL-C (mmol/L), n (%)	
< 2.59	29,822 (61.1)
≥2.59	11,817 (24.2)
≥3.37	5004 (10.3)
≥4.14	1495 (3.1)
≥4.92	632 (1.3)
TG (mmol/L), n (%)	
< 1.70	31,502 (64.6)
≥1.70	7616 (15.6)
≥2.26	8237 (16.9)
≥5.65	1416 (2.9)
HDL-C (mmol/L), n (%)	
Men < 1.04; women < 1.30	24,427 (50.1)
Men ≥1.04; women ≥1.30	18,535 (38.0)
Men ≥1.55; women ≥1.81	5809 (11.9)
Non-HDL-C (mmol/L), n (%)	
< 3.37	31,465 (64.5)
≥3.37	9537 (19.6)
≥4.14	4607 (9.4)
≥4.92	3161 (6.5)

*Abbreviations: SD* standard deviation, *TC* total cholesterol, *LDL-C* low-density lipoprotein cholesterol, *TG* triglyceride, *HDL-C* high-density lipoprotein cholesterol, *non-HDL-C* non-high-density lipoprotein cholesterol

Only 29.4% of the total 56,784 diabetic inpatients had lipid-lowering drugs records. The proportions of inpatients who had records of statin and fibrate use were 27.3 and 2.8%, respectively. Of the inpatients with LDL-C ≥ 2.59 mmol/L, only 29.4% of inpatients had statin use records. Of the inpatients with TG ≥1.70 mmol/L, only 7.7% of inpatients had fibrate use records (Additional file 1: Table S1).

### The lipid profiles of inpatients with diabetes in each gender and age group

The lipid profiles of inpatients with diabetes in each gender group were summarized in Table 2. The proportions of men with LDL-C < 2.59 mmol/L, TG < 1.70 mmol/L, HDL-C ≥ 1.04 mmol/L (men) or ≥ 1.30 mmol/L (women) and non-HDL-C < 3.37 mmol/L were 63.8, 67.2, 54.4 and 66.9%, respectively. While the proportions of women were 56.9, 60.5, 42.8 and 60.8%, respectively. According to the NCEP-ATP III guideline, the proportions of men with optimal LDL-C, TG, HDL-C and non-HDL-C levels were higher than those of women.

**Table 2:** The lipid profiles between men and women with diabetes

Lipid profiles (mmol/L)	Gender		*p* ^†^
	Men	Women	
LDL-C, n (%)			< 0.001
< 2.59	19,057 (63.8)	10,765 (56.9)	
≥2.59	6941 (23.2)	4876 (25.8)	
≥3.37	2776 (9.3)	2228 (11.8)	
≥4.14	764 (2.6)	731 (3.9)	
≥4.92	328 (1.1)	304 (1.6)	
TG, n (%)			< 0.001
< 1.70	20,065 (67.2)	11,437 (60.5)	
≥1.70	4323 (14.5)	3293 (17.4)	
≥2.26	4581 (15.3)	3656 (19.3)	
≥5.65	898 (3.0)	518 (2.7)	
HDL-C, n (%)			< 0.001
Men < 1.04; women < 1.30	13,619 (45.6)	10,808 (57.2)	
Men ≥1.04; women ≥1.30	12,521 (41.9)	6014 (31.8)	
Men ≥1.55; women ≥1.81	3727 (12.5)	2082 (11.0)	
Non-HDL-C, n (%)			< 0.001
< 3.37	19,973 (66.9)	11,492 (60.8)	
≥3.37	5587 (18.7)	3950 (20.9)	
≥4.14	2577 (8.6)	2030 (10.7)	
≥4.92	1730 (5.8)	1431 (7.6)	

*Abbreviations: LDL-C* low-density lipoprotein cholesterol, *TG* triglyceride, *HDL-C* high-density lipoprotein cholesterol, *non-HDL-C* non-high-density lipoprotein cholesterol

^†^Chi-square tests of proportions

The lipid profiles of inpatients with diabetes in each age group were displayed in Table 3. Overall, the proportions of optimal LDL-C, TG, HDL-C and non-HDL-C levels in the older age groups were higher than those in the younger age groups. Inpatients in the 46 to 64 years old group had the lowest proportion (56.8%) of optimal LDL-C level when compared with inpatients in the other age groups.

**Table 3:** The lipid profiles of inpatients with diabetes in each age group

Lipid profiles (mmol/L)	Age group (years)				
	≤45	46–64	65–79	≥80	*p* ^†^
LDL-C, n (%)					< 0.001
< 2.59	2721 (58.8)	11,035 (56.8)	12,255 (63.4)	3811 (70.7)	
≥2.59	1107 (23.9)	5105 (26.3)	4543 (23.5)	1062 (19.7)	
≥3.37	532 (11.5)	2286 (11.8)	1823 (9.4)	363 (6.7)	
≥4.14	160 (3.5)	680 (3.5)	526 (2.7)	129 (2.4)	
≥4.92	107 (2.3)	327 (1.7)	172 (0.9)	26 (0.5)	
TG, n (%)					< 0.001
< 1.70	2131 (46.0)	11,817 (60.8)	13,408 (69.4)	4146 (76.9)	
≥1.70	767 (16.6)	3174 (16.3)	2986 (15.5)	689 (12.8)	
≥2.26	1211 (26.2)	3834 (19.7)	2674 (13.8)	518 (9.6)	
≥5.65	519 (11.2)	608 (3.1)	251 (1.3)	38 (0.7)	
HDL-C, n (%)					< 0.001
Men < 1.04; women < 1.30	2866 (61.9)	9984 (51.4)	9222 (47.7)	2355 (43.7)	
Men ≥1.04; women ≥1.30	1400 (30.3)	7327 (37.7)	7599 (39.3)	2209 (41.0)	
Men ≥1.55; women ≥1.81	362 (7.8)	2122 (10.9)	2498 (12.9)	827 (15.3)	
Non-HDL-C, n (%)					< 0.001
< 3.37	2406 (52.0)	11,627 (59.8)	13,292 (68.8)	4140 (76.8)	
≥3.37	977 (21.1)	4173 (21.5)	3608 (18.7)	779 (14.5)	
≥4.14	580 (12.5)	2198 (11.3)	1519 (7.9)	310 (5.8)	
≥4.92	665 (14.4)	1435 (7.4)	899 (4.7)	162 (3.0)	

*Abbreviations: LDL-C* low-density lipoprotein cholesterol, *TG* triglyceride, *HDL-C* high-density lipoprotein cholesterol, *non-HDL-C* non-high-density lipoprotein cholesterol

^†^Chi-square tests of proportions

### The lipid profiles of diabetic inpatients with different cardiocerebrovascular diseases

**Fig. 1:**
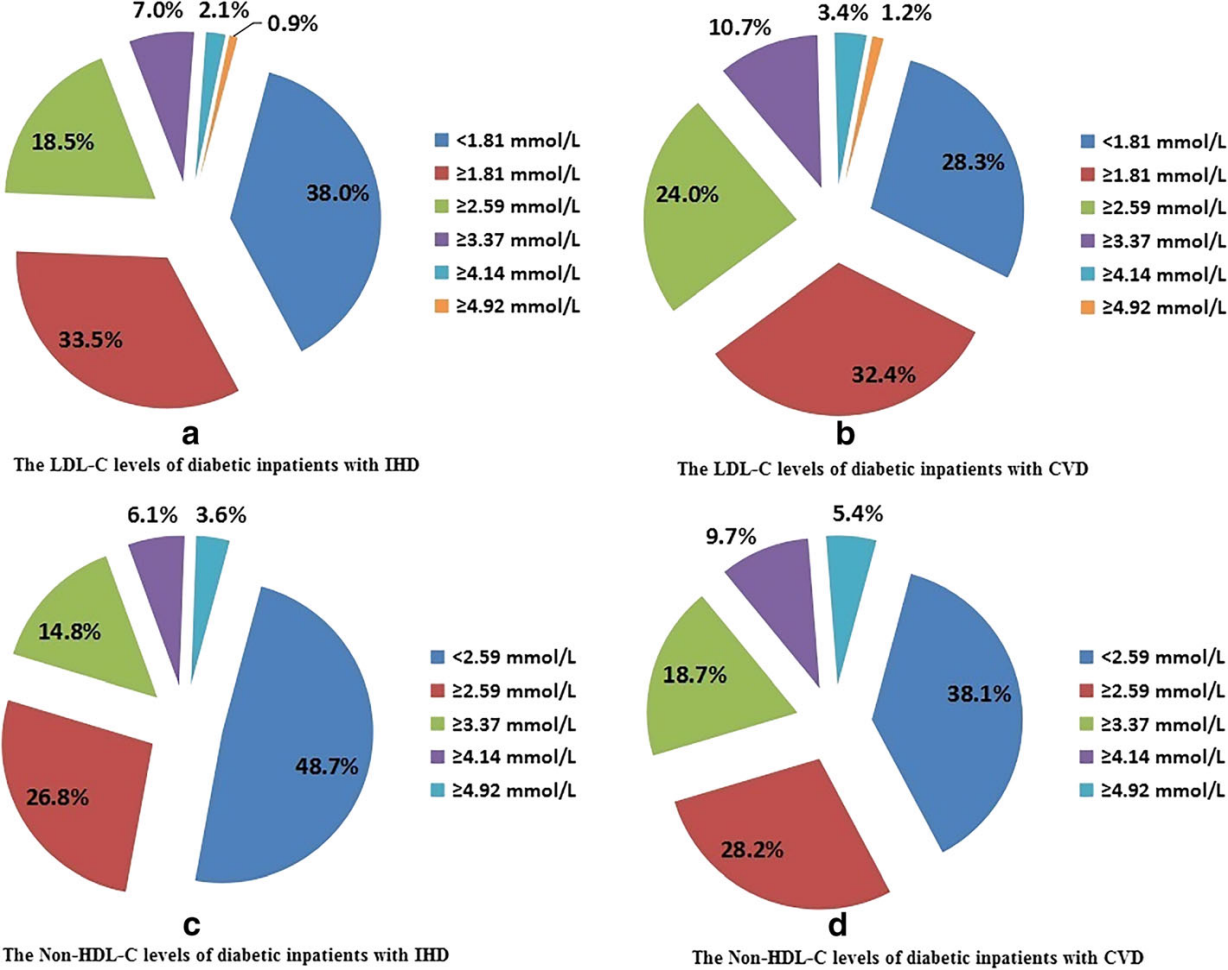
The LDL-C and non-HDL-C levels of diabetic inpatients with IHD and CVD. LDL-C = low-density lipoprotein cholesterol, non-HDL-C = non-high-density lipoprotein cholesterol, IHD = ischemic heart disease, CVD = cerebrovascular disease

There were 7762 (13.7%) inpatients with IHD and 8201 (14.4%) inpatients with CVD, respectively. The LDL-C and non-HDL-C levels of diabetic inpatients with IHD and CVD are displayed in Fig. 1. Of diabetic inpatients with IHD, there were 38.0% of them with LDL-C < 1.81 mmol/L. Of diabetic inpatients with CVD, the proportion was lower (28.3%). A total of 48.7% diabetic inpatients with IHD and 38.1% diabetic inpatients with CVD controlled their non-HDL-C levels below 2.59 mmol/L. About 66.3% diabetic inpatients with IDH and 64.8% diabetic inpatients with CVD controlled their TG levels below 1.70 mmol/L. Nearly half of diabetic inpatients with IHD (48.1%) and CVD (49.9%) had optimal HDL-C levels.

### Related factors associated with LDL-C, TG, HDL-C and non-HDL-C at optimal levels

Table 4 describes the related factors for lipids at optimal levels by multivariate logistic regression analyses. Women were less likely to have optimal levels of LDL-C, TG, HDL-C and non-HDL-C than men after adjustment for age, history of smoking and alchohol, CKD and lipid-lowering drugs. On the whole, older inpatients were more likely to obtain optimal levels of LDL-C, TG, HDL-C and non-HDL-C than younger patients after adjusting for gender, history of smoking and alchohol, CKD and lipid-lowering drugs. However, inpatients between 46 and 64 years old were least likely to obtain optimal LDL-C levels. Inpatients with CKD were more likely to obtain optimal levels of LDL-C and were less likely to obtain optimal levels of TG and HDL-C after adjusting for gender, age, history of smoking and alchohol and lipid-lowering drugs.

**Table 4:** The related factors for lipids at optimal levels by multivariate logistic regression analysis

Variables	LDL-C at optimal level OR (95% CI)	TG at optimal level OR (95% CI)	HDL-C at optimal level OR (95% CI)	Non-HDL-C at optimal level OR (95% CI)
Gender				
Men	1	1	1	1
Women	0.795 (0.758–0.835)^*^	0.677 (0.643–0.712)^*^	0.549 (0.524–0.576)^*^	0.729 (0.694–0.767)^*^
Age (years)				
≤45	1	1	1	1
46–64	0.915 (0.855–0.979)^**^	2.008 (1.876–2.149)^*^	1.690 (1.577–1.810)^*^	1.421 (1.329–1.520)^*^
65–79	1.212 (1.131–1.298)^*^	3.357 (3.128–3.602)^*^	2.047 (1.908–2.195)^*^	2.173 (2.028–2.329)^*^
≥80	1.577 (1.444–1.721)^*^	5.198 (4.738–5.703)^*^	2.361 (2.167–2.572)^*^	3.166 (2.892–3.467)^*^
History of smoking				
No	1	1	1	1
Yes	1.089 (1.034–1.146)^*^	0.994 (0.942–1.049)^***^	0.934 (0.889–0.981)^**^	1.005 (0.954–1.060)^***^
History of alchohol				
No	1	1	1	1
Yes	1.028 (0.977–1.082)^***^	0.952 (0.903–1.004)^***^	0.867 (0.825–0.911)^*^	0.965 (0.916–1.017)^***^
CKD				
No	1	1	1	1
Yes	1.184 (1.132–1.239)^*^	0.723 (0.690–0.757)^*^	0.849 (0.813–0.887)^*^	1.045 (0.998–1.095)^***^
Lipid-lowering drugs				
No	1	1	1	1
Yes	0.973 (0.935–1.014)^***^	0.528 (0.506–0.550)^*^	0.951 (0.915–0.990)^**^	0.786 (0.755–0.819)^*^

*Abbreviations: LDL-C* low-density lipoprotein cholesterol, *TG* triglyceride, *HDL-C* high-density lipoprotein cholesterol, *non-HDL-C* non-high-density lipoprotein cholesterol, *OR* odds ratio, *CI* confidence interval, *CKD* chronic kidney disease

^*^p ≤ 0.001; ^**^p ≤ 0.01; ^***^p > 0.05

## Discussion

This study has revealed that although most diabetic inpatients from a public university-affiliated tertiary hospital in southwest China have lipid measurements, the control of dyslipidemia in diabetic inpatients is not good enough.

Our data showed that 85.9% diabetic inpatients had lipid tests. According to the ADA guideline for the management of diabetes, it is appropriate to examine blood lipids at least once a year for most diabetic patients. And it is acceptable to examine blood lipids twice a year for diabetic patients without dyslipidemia [10]. Our data displayed that the proportions of diabetic inpatients with optimal levels of LDL-C and non-HDL-C were 61.1 and 64.5%, respectively. The US National Health and Nutrition Examination Survey 1999–2000 report showed that 25.3% subjects with diabetes were out of control for LDL-C (> 2.59 mmol/L) [11]. Another American registry study demonstrated that among the outpatients with diabetes, 73.9% of patients controlled their LDL-C levels below 2.59 mmol/L and 72.0% of patients controlled their non-HDL-C levels below 3.37 mmol/L. A total of 68.3% patients achieved both LDL-C and non-HDL-C goals [12]. In the current study, the proportions of diabetic inpatients achieved HDL-C and TG goals were 49.9 and 64.6%, respectively. A multicenter, non-interventional, cross-sectional study from India revealed that the control rates of HDL-C (men ≥1.04 mmol/L; women ≥1.30 mmol/L) and TG (< 1.70 mmol/L) were 60.48 and 57.54%, respectively [13]. All these data indicate that the control of dyslipidemia in diabetic patients is not good enough and there is still a gap between the recommendations of clinical guidelines and the real situation of clinical practice in achieving lipid level goals. Clinicians have a long way to go to narrow down this gap.

In this study, the control rates of LDL-C and non-HDL-C levels in diabetic inpatients with IHD were 38.0 and 48.7%, respectively. Which were much lower in diabetic inpatients with CVD (28.3 and 38.1%, respectively). Our findings were consistent with the previous studies. Mithal et al. showed that only 22.87% of diabetic patients with overt cardiovascular diseases reached LDL-C goal (< 70 mg/dL) across India [13]. This proportion was even much lower in Kennady et al’s study, in which only 15% of the high-risk population attained LDL-C level < 70 mg/dL [14]. LDL-C is the main target in the prevention of cardiovascular events. A meta-analysis including 90,056 participants from 14 randomized trials revealed that 1 mmol/L (40 mg/dL) reduction in LDL-C level can reduce all-cause mortality by 12% and coronary mortality by 19%. [15]. Non-HDL-C is another indicator of cardiovascular death. Data from the Lipid Research Program Follow-up Study showed that an increase of 0.78 mmol/L (30 mg/dL) in non-HDL-C level can increase CVD risk by 19% in men and 11% in women, respectively [16]. All these data suggest that the lipid control of diabetic patients with cardiocerebrovascular diseases is far from sufficient and the lipid management of diabetic patients at high cardiocerebrovascular risk should be strengthened.

Female diabetic inpatients were less likely to reach optimal levels of LDL-C, TG, HDL-C and non-HDL-C than male diabetic inpatients after adjusting for potential confounding factors in this study. Our findings are consistent with previous studies which revealed gender disparities in lipid control did exist. Zhang et al. demonstrated that the mean LDL-C, TC and TG levels in diabetic women with concomitant CHD were higher than those in diabetic men. And women were less likely to achieve their LDL-C target irrespective of age [17]. Li et al. revealed that TC and LDL-C levels in female outpatients were significantly higher than those in male outpatients [18]. The causes for the gender imbalance in lipid control are not very clear. One possible explanation is that women may have lower adherence to statins due to muscular symptoms when compared with men [19]. Another point worth noting is that the type and dose of lipid-lowering drugs were not adjusted in the current study, which can influence the lipid levels. All these findings suggest that the lipid control in female diabetic patients should be strengthened and individualized lipid management strategies taking gender disparities into consideration should be established. And further research is needed to investigate the underlying mechanism for poorer lipid control among diabetic women.

Older diabetic inpatients were more likely to obtain optimal LDL-C, TG, HDL-C and non-HDL-C levels in our study. And similar results were found in Spinler et al’s study [12]. In the current study, we should also note that patients aged 46 to 64 years old had the lowest proportion of optimal LDL-C level after adjusting for gender, history of alchohol and smoking, CKD and lipid-lowering drugs.

Inpatients with CKD were more likely to obtain optimal levels of LDL-C and were less likely to obtain optimal levels of TG and HDL-C in this study. Dyslipidemia is very common in CKD patients and usually varies with the renal function. As kidney function declines, TG levels tend to increase and HDL-C levels decline. Whereas there is no obvious difference in the LDL-C levels between stages 1 to 4 CKD and the general population [20, 21]. The impact of renal function on lipid control deserves further research.

This study may have several potential limitations. First, this is a cross-sectional study. Only correlations rather than causal relationships can be established due to the study design. The collection of data is also retrospective and therefore the recall bias can’t be totally ruled out. So this study could not determine the exact reasons for poor lipid control in diabetic patients. Second, this is a single center study and all the participants are from the same tertiary hospital in southwest China, so we may not generalize the results to other demographic groups. Further multi-centered, prospective cohort studies are needed to investigate the lipid control in different populations. Third, the intensity of lipid-lowering drugs and medication adherence is not available in this study. Therefore, we can’t adjust the unmeasured confounding factors.

## Conclusions

In conclusion, the lipid control in diabetic inpatients in southwest China was not good enough. Individualized strategies of lipid management should be taken to bridge the gap between the recommendations of clinical guidelines and the real situation of clinical practice. The results of this study are of value for the lipid management of diabetic inpatients in China, while further multi-centered, prospective cohort studies are needed to investigate the lipid control in different populations.

## Additional file

Additional file 1: **Table S1.** The status of lipid-lowering therapy of diabetic inpatients. (DOCX 13 kb)

## Abbreviations

ADA: American diabetes association
CHD: Coronary heart disease
CI: Confidence interval
CKD: Chronic kidney disease
CVD: Cerebrovascular disease
eGFR: Estimated glomerular filtration rate
EMR: Electronic medical record
HDL-C: High-density lipoprotein cholesterol
ICD-10: International classification of diseases 10th revision
IDF: International diabetes federation
IHD: Ischemic heart disease
LDL-C: Low-density lipoprotein cholesterol
NCEP-ATP III: National cholesterol education program-adult treatment panel III
non-HDL-C: Non-high-density lipoprotein cholesterol
OR: Odds ratio
SD: Standard deviation
TC: Total cholesterol
TG: Triglyceride
