# Supplementary material for: Characteristics of lipid profiles and lipid control in patients with diabetes in a tertiary hospital in Southwest China: an observational study based on electronic medical records
Source: Lipids Health Dis. 2019 Jan 12;18:13. doi: 10.1186/s12944-018-0945-8 (PMC6330454; doi:10.1186/s12944-018-0945-8)
Supplement: Supplementary file 1 — Table S1. The status of lipid-lowering therapy of diabetic inpatients. (DOCX 13 kb) [file 12944_2018_945_MOESM1_ESM.docx]

**Additional file 1: Table S1.** The status of lipid-lowering therapy of diabetic inpatients

| **Status of lipid-lowering therapy** | **Number (percentage)** |
| --- | --- |
| [Lipid-lowering](D:/%E8%BD%AF%E4%BB%B6%E5%AE%89%E8%A3%85/%E6%9C%89%E9%81%93%E8%AF%8D%E5%85%B8/Dict/7.5.2.0/resultui/dict/?keyword=lipid-lowering)[drugs](D:/%E8%BD%AF%E4%BB%B6%E5%AE%89%E8%A3%85/%E6%9C%89%E9%81%93%E8%AF%8D%E5%85%B8/Dict/7.5.2.0/resultui/dict/?keyword=drugs) records |  |
| No | 40115 (70.6) |
| Yes | 16669 (29.4) |
| Statin use records |  |
| No | 41285 (72.7) |
| Yes | 15499 (27.3) |
| Fibrate use records |  |
| No | 55221 (97.2) |
| Yes | 1563 (2.8) |
| Total | 56784 (100.0) |
